# Beyond Clotting: A Role of Platelets in CNS Repair?

**DOI:** 10.3389/fncel.2015.00511

**Published:** 2016-01-20

**Authors:** Francisco J. Rivera, Ilias Kazanis, Cedric Ghevaert, Ludwig Aigner

**Affiliations:** ^1^Institute of Molecular Regenerative Medicine, Paracelsus Medical University SalzburgSalzburg, Austria; ^2^Spinal Cord Injury and Tissue Regeneration Center Salzburg, Paracelsus Medical University SalzburgSalzburg, Austria; ^3^Department of Clinical Neuroscience, Wellcome Trust-MRC Cambridge Stem Cell Institute, University of CambridgeCambridge, UK; ^4^Department of Biology, University of PatrasPatras, Greece; ^5^Department of Haematology, University of CambridgeCambridge, UK; ^6^National Health Service Blood and Transplant, Cambridge Biomedical CampusCambridge, UK

**Keywords:** haemostasis, inflammation, angiogenesis, stroke, demyelination, neural stem/progenitor cells, CNS regeneration

## “Non-canonic” roles of platelets: inflammation, angiogenesis and tissue regeneration

Platelets are small, oval, circulating, anucleate cells that upon endothelial damage form a haemostatic plug and stop blood leakage. Circulating platelets derive from bone-marrow-resident megakaryocytes that daily produce and release approximately 100 billions of new platelets into the blood stream (Kaushansky, 2006; Semple et al., 2011). During haemostasis, tethering platelets adhere to the vascular injury through the interaction between their glycoprotein (GP) Ib/V/IX receptor complex and GP VI/GP Ia with the von Willebrand factor (vWF) and collagen provided by the lesioned environment, respectively. Adherent platelets aggregate and secrete platelet activation mediators, such as Thromboxane A2 (TXA2) and adenosine diphosphate (ADP). After activation, the platelets membrane surface becomes procoagulant enhancing the coagulation cascade ending in the formation and stabilization of the haemostatic plug and arresting blood leakage.

Platelet function is not restricted to haemostasis, as platelets also have inflammatory, angiogenic, and tissue repair properties (Nurden, 2011). Within their storage compartments (α-granules and dense granules), platelets store a plethora of bioactive molecules that, under specific circumstances, are secreted to the extracellular space targeting other cell types. Platelets-derived molecules include proteins such as chemokines, cytokines, and growth factors, as well as RNAs and microparticles (Brill et al., 2005; Chen et al., 2012; Lohmann et al., 2012; Schallmoser and Strunk, 2013; Warnke et al., 2013). Platelets granules contain several pro-inflammatory and anti-inflammatory molecules that contribute to immunity. In fact, platelets react against pathogens and regulate immune cells function (reviewed in Semple et al., 2011). For example, during inflammation, GPIbα, and P-selectin located at the surface of platelets interact with PSGL-1 and Mac-1 on monocytes/macrophages inducing their recruitment and activation (reviwed in Gawaz et al., 2005). Also, CD154 in activated platelets (Henn et al., 1998) binds to CD40 on endothelial cells (ECs) inducing the expression of cell adhesion molecules (i.e., VCAM1, ICAM1) and the endothelial release of CC-chemokine ligand 2 (CCL2) promoting the leukocyte recruitment to inflammatory sites (Andre et al., 2002). Moreover, CD154 supports B cell differentiation (Elzey et al., 2003; Von Hundelshausen and Weber, 2007) and platelet-secreted transforming growth factor beta (TGF-β) controls T_reg_ cell differentiation (Tran, 2012) indicating that the contribution of platelets to immunity is not restricted to the innate system but also involves adaptive response.

Platelets apparently also shape angiogenesis, which is a complex process that consists in the formation/sprouting of new capillaries from preexisting vessels. Platelets have a dual role. First, they stimulate ECs proliferation and can promote capillary formation (Pipili-Synetos et al., 1998). Indeed, α-granules contain several pro-angiogenic molecules that are secreted upon the activation of platelets, such as vascular endothelial growth factor (VEGF), platelet-derived growth factor (PDGF), TGF-β, basic fibroblast growth factor (bFGF), epidermal growth factor (EGF), sphingosine-1-phosphate (S1P), etc (Kaplan et al., 1979; Heldin et al., 1981; Nakamura et al., 1985; Folkman and Klagsbrun, 1987; Ben-Ezra et al., 1990; Mohle et al., 1997; English et al., 2000; Jonnalagadda et al., 2014). Second, platelets are required to avoid leakage from angiogenic vessels and their absence inhibits the formation of new vessels *in vivo* (Kisucka et al., 2006).

Haemostasis, inflammation and angiogenesis are essential processes for tissue repair; thus, platelets are critically involved in many mechanisms that operate along the healing process (reviewed in Gurtner et al., 2008; Gawaz and Vogel, 2013). Upon tissue damage provoked by trauma or local ischemia, circulating platelets accumulate and become activated at the lesion site releasing their bioactive molecules into the damaged microenvironment and contributing to tissue repair and regeneration. For instance, stromal cell-derived factor-1 (SDF-1), hepatocyte growth factor (HGF), PDGF, serotonin, ADP, and platelets-derived microparticles regulate recruitment, proliferation, survival, and differentiation not only of immune cells (neutrophils, monocytes) necessary for the local inflammatory and the phagocytic responses, but also of cells that directly repair the lesion such as fibroblast, smooth muscle cells and tissue-specific progenitor cells (Nakamura et al., 1986; Crowley et al., 1994; Stellos et al., 2008, 2010; Mazzucco et al., 2010).

## Platelets influence CNS inflammation: impact on repair?

Regardless of its immune privileged condition and the presence of the blood-brain-barrier (BBB), the CNS is not free from the action of platelets, particularly, in response to injury. As expected, after their adherence to endothelial cells, platelets activate, and recruit leukocytes into the damaged CNS tissue (Simon, 2012; Langer and Chavakis, 2013), thus, platelets interact with different cells in the neurovascular niche including neurons, glial cells, endothelial cells, pericytes, and other blood-derived cells (Hayon et al., 2013; Sotnikov et al., 2013). This particular feature confers platelets a substantial role in CNS inflammation in different pathological scenarios. After stroke, platelets adhere to the endothelium and get activated provoking further thrombo-inflammatory events exaggerating infarct development (Kleinschnitz et al., 2007; Nieswandt et al., 2011). In Alzheimer's disease (AD) the BBB is partially leaky and vascular inflammation occurs (Sardi et al., 2011). Interestingly, platelets might be contributing to the propagation of AD as they carry amyloid precursor protein and the amyloid beta, two peptides that are found around vessels in AD patients that constitute one of the molecular mechanisms for AD pathogenesis (Skovronsky et al., 2001; Catricala et al., 2012). In multiple sclerosis (MS), an autoimmune CNS demyelinating disease, platelets also seem to be involved to the pathology since they have been found in human chronic active MS lesions (Lock et al., 2002; Langer et al., 2012; Steinman, 2012). In an animal model for MS, platelets promote leukocyte infiltration as well as CNS inflammation (Lock et al., 2002; Langer et al., 2012). Therefore, platelets contribute to neuroinflammation and an altered platelet functions may lead to pathological conditions.

Besides their role in pathogenesis, platelets might also be involved in the regulation of regenerative processes by interacting with CNS stem/progenitor cells. Adult neural stem cells (NSCs) are undifferentiated self-renewing multipotent cells that reside in the subgranular zone (SGZ) in the dentate gyrus of the hippocampus and in the subventricular zone (SVZ) of the wall of the lateral ventricles (Altman, 1965; Gage, 2000; Alvarez-Buylla and Garcia-Verdugo, 2002). Oligodendrocyte progenitor cells (OPCs) represent the major cellular source for remyelinating oligodendrocytes and are widely spread throughout the CNS (Ffrench-Constant and Raff, 1986; Woodruff and Franklin, 1999; Franklin and Ffrench-Constant, 2008). Interestingly, NSCs and OPCs drive CNS repair in response to stroke or to MS-associated demyelination (Arvidsson et al., 2002; Franklin, 2002; Kokaia and Lindvall, 2003; Gonzalez-Perez et al., 2009). While neuroinflammation was mainly considered to be an aggravating factor, several recent studies have revealed a supportive role in CNS repair (Patel et al., 2010; Jaerve and Muller, 2012; Miron and Franklin, 2014). For example, the very complex process of remyelination (Franklin and Ffrench-Constant, 2008; Rivera et al., 2010) involves a crucial inflammatory stage that precedes regeneration and occurs acutely after myelin damage. This innate immune response is, at least partially, mediated by blood-recruited macrophages and CNS-resident microglial cells. During remyelination, circulating monocytes/macrophages are recruited by chemotaxis (Charo and Ransohoff, 2006; Ruckh et al., 2012) and, depending on their inflammatory state, exert the following functional roles: (i) they are responsible for the removal (clearance) of myelin debris [which is a potent inhibitor of OPC differentiation (Kotter et al., 2006; Baer et al., 2009)] through phagocytosis, and (ii) they secrete cytokines, growth- and neurotrophic factors that stimulate OPC responses to demyelination (Setzu et al., 2006; Zhao et al., 2006; Ruckh et al., 2012; Miron et al., 2013). A regulation of macrophage recruitment and activity is essential to couple inflammation and regeneration during CNS myelin repair (Miron and Franklin, 2014). However, the cellular and molecular cues that regulate these events are still unknown. As previously mentioned, platelets promote the endothelial secretion of chemokines known to recruit circulating monocytes/macrophages (Gawaz et al., 1998, 2000). Furthermore, through the secretion of PDGF and platelet factor 4 (PF-4), activated platelets directly promote the recruitment of monocytes and modulate their activity (Deuel et al., 1982; Brandt et al., 2000; Fricke et al., 2004). Thus, it can be hypothesized that circulating platelets might influence macrophage/microglia recruitment and activity thereby linking neuroinflammation to CNS repair.

## Platelets modulate CNS-resident stem/progenitor cell function: impact on repair?

Supporting the previous hypothesis, a series of findings suggest that platelets directly exert CNS-regenerative activities and might contribute to neuroregeneration (see Table 1). Recently we reported that upon demyelination in the corpus callosum (CC), platelets specifically accumulate within the ipsilateral SVZ vasculature, a process associated with an enhanced survival of SVZ-resident NSCs (Kazanis et al., 2015). Importantly, we found that a mechanical non-demyelinating lesion within the CC is not enough to induce such accumulation of platelets in the SVZ vasculature, indicating that cellular degeneration is required for such an effect. Considering that SVZ-derived NSCs contribute to remyelination in the CC (Jablonska et al., 2010; Xing et al., 2014), these findings suggest that platelets might play a role in controlling the NSCs pool available for CNS repair. The mechanisms that mediate the very specific accumulation of platelets in the SVZ vasculature far from the lesion site, and that promote NSCs survival are not known. However, platelets derived molecules might be involved as in the same study we found that platelet lysate (PL) protects proliferating NSCs from apoptosis (Kazanis et al., 2015). Furthermore, it has been previously suggested that activated platelets contribute to recovery after brain injury (Hayon et al., 2012c). For example, it has been shown in an animal model for stroke that infused platelets derived microparticles (PMP) increased cell proliferation, neurogenesis and angiogenesis at the infarct boundary zone leading to improvements in behavioral outcomes (Hayon et al., 2012a). In addition to this, a different study showed that PMP promotes NSCs survival and increased their differentiation potential to glia and neurons (Hayon et al., 2012b). Also, upon intracerebroventricular administration of PL into an experimental model of stroke resulted in a significant increase in angiogenesis and in the number of proliferating SVZ-resident NSCs (Hayon et al., 2013). Besides these findings, several platelets derived molecules influence CNS progenitor function (see Table 1). For instance, the dense granules of platelets contain serotonin (White, 1968), which is known to control NSCs activity and adult neurogenesis (Brezun and Daszuta, 1999; Banasr et al., 2004; Goto et al., 2015). The effect of platelets derived molecules might not only target NSCs but also OPCs during remyelination. For instance, platelets' α-granules contain large quantities of PDGF and bFGF (Lohmann et al., 2012; Schallmoser and Strunk, 2013), factors that are known to promote OPCs survival, proliferation, and recruitment (Woodruff et al., 2004; Murtie et al., 2005; Zhou et al., 2006). Moreover, upon activation, platelets' α-granules secrete S1P (English et al., 2000; Jonnalagadda et al., 2014), a molecule, that is known to modulate OPCs survival, proliferation, and differentiation (Jung et al., 2007). In summary, platelets react to injury and secrete a plethora of bioactive molecules that might directly influence NSCs and OPCs function, probably, modulating CNS repair. This hypothesis could be evaluated by studying neuroregeneration in animal models that display platelet deficiencies (number and/or function). Also, by exploring gene expression databases complemented with proteomics data, further studies could identify molecules contained in platelets that may influence CNS repair.

**Table 1:** **Evidences suggesting possible direct contribution of platelets in CNS repair**.

**Platelets derived activity or factor**	**Experimental Model**	**Findings**	**References**
Circulating Platelets	Lysolecithin-induced demyelination in the CC	Platelets aggregation in the SVZ vasculature associated to NSCs survival	Kazanis et al., 2015
Platelet lysate (PL)	*In vitro* proliferating NSCs	PL promotes NSCs survival and protects from apoptosis	Kazanis et al., 2015
Platelets-derived microparticles (PMP)	Permanent middle cerebral artery occlusion (PMCAO). PMP administrated to the brain surface	PMP increases cell proliferation, neurogenesis, and angiogenesis	Hayon et al., 2012a
PMP	*In vitro* NSCs	PMP promotes survival and increases the differentiation potential of NSCs	Hayon et al., 2012b
PL	PMCAO. PL administrated into the lateral ventricles	PL increases the number of NSC and angiogenesis in the subventricular zone (SVZ) as well as in the peri-lesion cortex	Hayon et al., 2013
Serotonin (contained in dense granules)	*In vivo* normal wild type rats. Systemic administration of serotonin	Serotonin regulate NSCs proliferation in the SVZ and hippocampus and modulate adult neurogenesis	Banasr et al., 2004
PDGF and bFGF (contained in α-granules)	Cuprizone-induced demyelination in PDGFαR+∕− mice, FGF2 knockout (−∕−) mice, and PDGFαR+∕− FGF2−∕− mice	PDGF and bFGF regulate OPCs proliferation and differentiation during CNS remyelination	Murtie et al., 2005
S1P (contained in α-granules)	*In vitro* OPCs. Use of S1P analogs: FTY720 and FTY720P, both modulators of S1P receptors	By different mechanisms S1P and its receptors regulate OPCs proliferation, survival, and differentiation	Jung et al., 2007

## Final remarks

There is accumulating evidence that the role of platelets is not restricted to haemostasis, but it also involves the regulation of inflammation, angiogenesis and tissue repair. The CNS contains NSCs and OPCs that contribute to cellular turnover and CNS repair. In light of the accumulating evidence that associates platelets to neuroinflammation, especially under pathological conditions, their potential role in CNS repair has to be further investigated. Recent findings indicate that neuroinflammation is also relevant for CNS repair as it contributes to debris clearance and controls CNS-resident stem/progenitor cells function, suggesting a potential role for platelets by linking inflammation to regeneration. This hypothesis is supported by the facts that circulating platelets react to CNS injury and accumulate within the adult stem cell niche and that activated platelets release a plethora of bioactive molecules that not only regulate immune cells activity but also directly modulates NSC and OPC respond to injury. It is, therefore, likely that platelets might modulate CNS repair. Prospectively, this specific lesion-induced accumulation of circulating platelets at sites of tissue damage, inflammation, and even stem/progenitor cell activity (as in Kazanis et al., 2015) opens the possibility to use genetically manipulated platelets or manufactured platelet-like particles (Risitano et al., 2012; Brown et al., 2014) when aiming for the delivery of specific molecules directly to targeted areas.

## Author contributions

FR wrote the manuscript. IK, CG, and LA critically reviewed the manuscript. All authors read and approved the final version of the manuscript.

### Conflict of interest statement

The authors declare that the research was conducted in the absence of any commercial or financial relationships that could be construed as a potential conflict of interest.
